# Metrological traceability in process analytical technologies and point-of-need technologies for food safety and quality control: not a straightforward issue

**DOI:** 10.1007/s00216-022-04398-5

**Published:** 2022-11-11

**Authors:** Monica Mattarozzi, Eleni Laski, Alessandro Bertucci, Marco Giannetto, Federica Bianchi, Claudia Zoani, Maria Careri

**Affiliations:** 1 Department of Chemistry, Life Sciences and Environmental Sustainability, University of Parma, Parco Area Delle Scienze 17/A, 43124 Parma, Italy; 2 Interdepartmental Centre SITEIA.PARMA, University of Parma, Technopole Pad 33 Parco Area Delle Scienze, 43124 Parma, Italy; 3 Interdepartmental Centre CIPACK, University of Parma, Technopole Pad 33 Parco Area Delle Scienze, 43124 Parma, Italy; 4 Department for Sustainability, Biotechnology and Agroindustry Division (SSPT-BIOAG), Casaccia Research Centre, Italian National Agency for New Technologies, Energy and Sustainable Economic Development (ENEA), Via Anguillarese 301, 00123 Rome, Italy

**Keywords:** Metrological traceability, Method validation, Rapid screening, Process analytical technology, Point of need, Food analysis

## Abstract

Traditional techniques for food analysis are based on off-line laboratory methods that are expensive and time-consuming and often require qualified personnel. Despite the high standards of accuracy and metrological traceability, these well-established methods do not facilitate real-time process monitoring and timely on-site decision-making as required for food safety and quality control. The future of food testing includes rapid, cost-effective, portable, and simple methods for both qualitative screening and quantification of food contaminants, as well as continuous, real-time measurement in production lines. Process automatization through process analytical technologies (PAT) is an increasing trend in the food industry as a way to achieve improved product quality, safety, and consistency, reduced production cycle times, minimal product waste or reworks, and the possibility for real-time product release. Novel methods of analysis for point-of-need (PON) screening could greatly improve food testing by allowing non-experts, such as consumers, to test in situ food products using portable instruments, smartphones, or even visual naked-eye inspections, or farmers and small producers to monitor products in the field. This requires the attention of the research community and devices manufacturers to ensure reliability of measurement results from PAT strategy and PON tests through the demonstration and critical evaluation of performance characteristics. The fitness for purpose of methods in real-life conditions is a priority that should not be overlooked in order to maintain an effective and harmonized food safety policy.

**Graphical Abstract:**

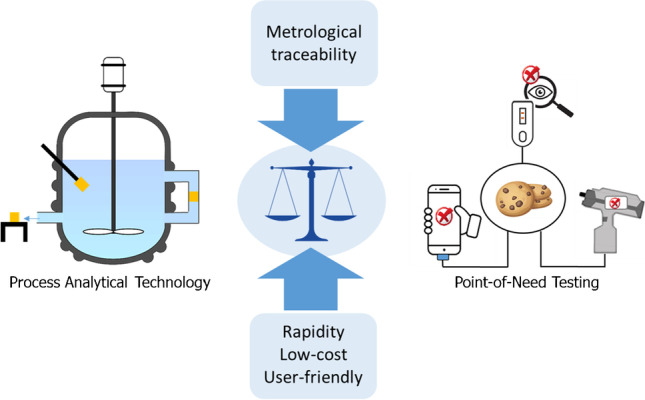

## Introduction

Currently, food quality and safety control still rely mainly on discontinuous laboratory-centralized analysis with traditional analytical methods involving sampling from production lines or stocks of raw materials and final products, or, at best, at-line measurements. Traditional methods for food testing are performed on benchtop instruments in a lab facility reserved for highly qualified personnel and are mainly based on spectroscopic, chromatographic, or mass spectrometry–based techniques. Although these off-line approaches can provide reliable, accurate, metrologically traceable, and comparable results [1], they cannot guarantee either in situ analysis or real-time snapshot of the production process in the food industry.

In this context, to fulfil the needs of the food industry, which is facing increasing demands for product yield, process efficiency, and high expectations for product quality and safety, a different approach is therefore required to improve the effectiveness of the food safety and quality management systems.

**Fig. 1:**
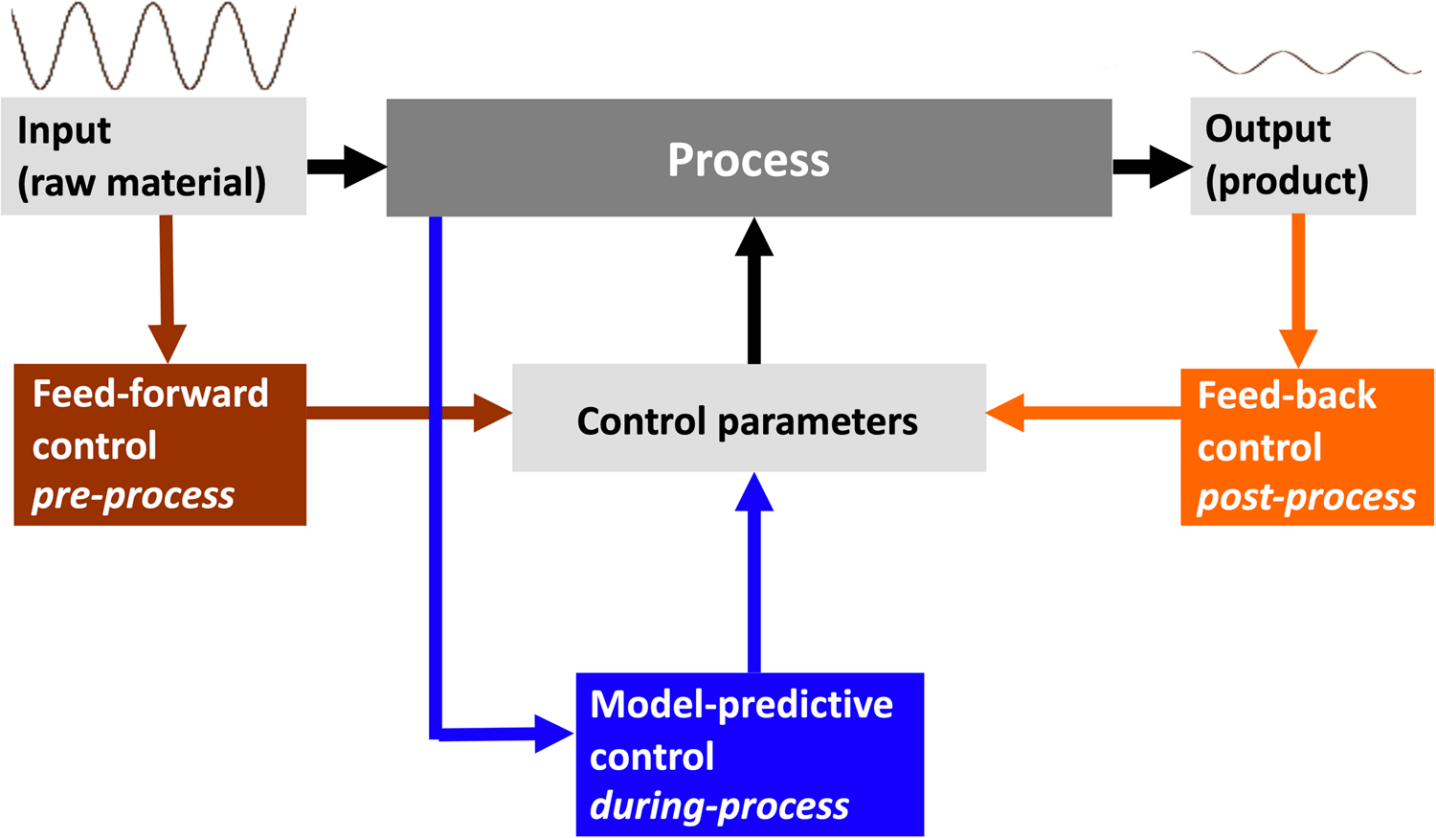
Schematic overview of process control strategies in food manufacturing. Reprinted with permission from [2]

The requirements in food quality and food safety might be addressed also through the use of rapid analysis methods and process analytical technology (PAT) [2, 3]. In 2004, the Food and Drug Administration defined PAT as a system to design, analyze, and control manufacturing processes through timely measurements of critical material attribute (CMA) and critical process parameters (CPP) which affect critical quality attributes (CQA). Although the PAT concept was initially derived from the pharmaceutical industry, the importance of product consistency, quality, and process automation has emerged also in the food industry, allowing to move analytics closer to the process for continuous production (Fig. 1) [2, 4, 5].

The integration of PAT in the food industry is closely linked to the concept of Industry 4.0, meaning applying digitalization and data exchange with the Internet of Things (IoT) and the networking of machines for process control automation [6–8].

The goal of real-time process monitoring is to ensure consistent final product quality and process efficiency by making the “quality by design” (QbD) approach possible, thus moving from a paradigm of “quality by testing”' to “building QbD.” To this aim, more extensive analytical data is needed, ideally obtained in-time on raw materials, process intermediates, and the quality attributes of the product.

Focusing on the issue of food safety, a European Union (EU) food strategy has been developed since the publication of the White Paper on Food Safety in 2000 [9], followed by the “General Food Law” (Regulation (EC) 178/2002), which laid down the general principles and requirements of food law in the EU and established the European Food Safety Authority (EFSA) and the Rapid Alert System for Food and Feed (RASFF) [10]. The role of the EU is also to assure the effectiveness of control systems at national level. This is the task of the Directorate-General for Health and Food Safety which carries out inspections in the EU Member States and in non-EU countries exporting to the EU aimed at monitoring compliance with EU legal obligations [1]. In the case of official controls on food, the EU requires the use of validated analytical methods, which provide accurate and metrologically traceable results [11]. In this context, EU Regulation 2017/625 assigns to European Union Reference Laboratories (EURLs) and National Reference Laboratories (NRLs) the role of maintaining the quality and reliability of analytical methods and results among the EU members, essential for a harmonized EU policy on food. In addition, the EU community promotes initiatives on scientific cooperation both to ensure reliable, comparable, and traceable analytical measurements, such as the METROFOOD-RI research infrastructure included in the ESFRI Roadmap for the domain Health and Food [12], and to establish a dialogue between the metrology community on food safety and stakeholders, such as Food-MetNet in the frame of EURAMET [13]. Such kind of initiatives highly promote applied metrology, which can be seen—along with scientific metrology and legal metrology—as one of the three main sub-fields of metrology in its definition of “science of measurement embracing both experimental and theoretical determinations at any level of uncertainty in any field of science and technology,” as defined by the International Bureau of Weights and Measures (BIPM, 2004). They can play a key role in enforcing the metrological infrastructure and supporting the application of metrological principles to real life and any practical application in different fields, including agrifood and food analysis. In particular, they might be seen as a bridge between the main international organizations (i.e., BIPM and OIML), standardization bodies (e.g., ISO, CEN, AOAC, Codex Alimentarius), and National Metrology Institutes, with the main actors of the food safety system and more in general the agrifood stakeholders, thus favoring to close the gaps related to metrological traceability of PAT strategies with reference to both their development and production from the research community and the manufacturers, and then their application in the food industry.

Recent analytical trends in the miniaturization of portable sensing devices combined with “smart” features enabled by wireless data sharing, on-line sampling systems, and analyzers, as well as in-line probes, have expanded the analytical panorama towards smart analytical devices [14] and brought innovation to in-process analysis interfaces. Regardless of the certain benefits offered by real-time process control and decentralized analysis, equal attention is required on assuring the robustness and constant accuracy of measurements considering that both analytical methods and analytical systems have to be tested to ensure that they meet the basic requirement to be fit for purpose [15].

Smart screening devices and testing kits, capable of providing qualitative or semi-quantitative information in a fast, cost-effective, and non-invasive way, are also increasingly being developed for on-site food analysis outside the laboratory and the process environment. Allowing for high-throughput analysis, screening tests are often chosen for routine large-scale food control when their purpose is just to obtain a rapid binary response to establish whether the sample is compliant or suspected non-compliant with respect to a defined concentration level [16]. Indeed, they can provide fast results on possible contaminations or non-conformity, thus reducing the number of samples to be submitted for regulatory testing [1]. In this context, rapid screening methods are not limited to food operators only, but emerging trends are even targeting consumers as end users. The so-called point-of-need (PON) or point-of-care (POC) testing with portable devices and disposable kits aims to be fast and simple, with minimum or no sample preparation and without requiring user expertise. As the analytical readout can be simplified to portable instruments, smartphones, and even naked-eye, usable also by non-experts, it is also important to ensure that the assay reading and the interpretation of the results do not cause additional uncertainty.

Considering the growing interest in real-time analysis and PAT systems for process control in the food industry, as well as the trend towards the development of smart devices for PON analysis of food products, this review paper critically points out the importance of demonstrating metrological traceability and reliability of the measurement results in real-life conditions, a challenge not easily met with the analytical tools of PAT and the analytical methods for PON testing. The need for rapid and cost-effective analysis should not outweigh the demand for reliable measurements for food quality and safety control.

## Process control through integrated PAT in food industry

PAT represents a revolution in industry, which is increasingly driving the shift from inferential monitoring and control of physical and engineering properties towards the real-time and continuous measurement of chemical parameters during the process itself for process monitoring and timely intervention in the presence of out-of-control conditions. PAT is based on the acquisition and consolidation of knowledge on process dynamics and mechanisms, followed by the implementation of automatic control strategies.

**Fig. 2:**
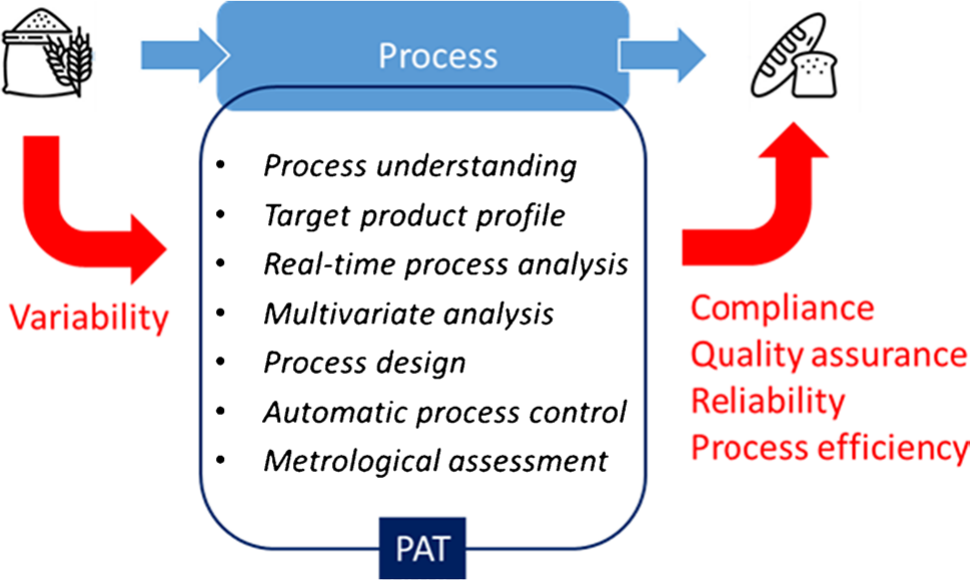
PAT tools and strategies to manage input variability of raw materials while ensuring product quality and process efficiency

The benefits for the food manufacturers from QbD in product design and achieving compliance can outweigh the investment in PAT, minimizing product losses, reducing waste and by-products, optimizing energy and raw material consumption, and maintaining consumers’ trust in food product safety and quality [4]. The food industry faces strict regulations and consumer demands for stringent quality, safety, and traceability controls, as well as high productivity of manufacturing facilities in a sustainable way. This has boosted food producers to develop and implement PAT strategies [2]. Process efficiency optimization and food quality assurance combined with minimizing the environmental footprint of food processing may be achieved by replacing recipe-based production and managing the variability of incoming raw materials during the process itself. PAT tools and related automatic control strategies allow process improvement by managing input variability that leads to improved efficiencies, better product quality, and enhanced consumer safety (Fig. 2).

In fact, the implementation of a PAT strategy in the food industry has to face the complexity and heterogeneity of food matrices, together with the different physical properties, the large chemical and biological variability of raw materials and process feedstocks, and complex food processing and hygiene requirements.

These conditions require rigorous criteria for representative sampling and assurance of the selectivity of the measurements. A univariate calibration model can only provide accurate results if the measured signal has no contributions from interfering compounds; otherwise, the results will be biased. Since no univariate method applied to spectroscopic data for process analysis has significant selectivity to monitor a target analyte without interferences from other species, multivariate data processing and calibration are needed to convert non-selective analyzer signals in selective information on the property of interest. In order to assure selectivity and good prediction ability of the empirical model, it is essential to choose a proper training set as well as a proper validation set to represent all the expected variations within which the model is applied. The compatibility of the materials used for the PAT sensors and probes with food is another aspect that must be taken into account [3].

### Process analyzers for PAT

Nowadays, a huge number of on-line/in-process analyzers and probes are being implemented and used in the food industry. Spectroscopy techniques like ultraviolet–visible (UV–Vis), mid-infrared (MID-IR), near-infrared (NIR), and Raman spectroscopy are the most widely used in PAT for chemical, physical, and rheological investigations, since they allow very fast (ms-s) and non-destructive measurements, require minimal or no sample preparation, enable remote analysis via fiber-optic probes and flow cells, allow multiplexing analysis, and provide the opportunity to assess several attributes simultaneously. The on-line analysis involves process materials being diverted from the main stream through a proper bypass, if necessary conditioned by a sampling system, and finally returned to the process stream or wasted; in the in-line approach, the measurements are performed directly in the process through immersion probes or non-contact mode [5, 17].

The implementation of PAT is further supported by the continuous evolution of chemometric tools applied to large and complex analytical data sets that generate classification and calibration models. These models must be properly validated to ensure good prediction performances. The development and maintenance of PAT require a synergy between well-defined analytical purposes and digitalization, networking, wireless communication, data transfer, big data, and artificial intelligence, which drive significant progress and improvements in process efficiency, profitability, and reliability [7]. Automatic electronic systems should support data acquisition and archiving, remote instrument control, development and execution of real-time chemometric models, data fusion from multisensor data, and instrument diagnostics to guarantee quality assurance towards digitized processes.

Recent reviews report the potential application of PAT in food production, such as the bakery industry [4], as well as in the processing of dairy products [5, 16]; however, there is still a long way to go for its establishment as a widespread tool for food process monitoring.

In this context, NIR spectroscopy together with chemometrics-based data analysis [18] owes much of its current popularity to research and development in the food sector, being one of the main analytical techniques applied to PAT in the food industry: low-cost miniaturized probes are currently available for transmission or reflection analysis, which can be easily incorporated into the processing line [19, 20]. Also, MID-IR spectroscopy [21, 22] as well as Vis–NIR [19, 23], Raman [24, 25], and fluorescence [26, 27] spectroscopy have shown promising results for potential application as PAT in the food industry. Other analytical tools for on-site quality control include electronic noses (e-noses) and electronic tongues (e-tongues), in which sensor arrays designed for untargeted analysis are used to obtain a fingerprint profile of the sample composition: however, it should be noted that their potential for PAT for timely decision-making during production has not yet been fully demonstrated. In most of the published research, e-noses are suggested as a rapid quality control tool for the detection of unsafe or deteriorating products in the post-harvest and storage phase [28–30], as well as process monitoring [20, 31]. E-tongues, mostly based on electrochemical sensors, have been evaluated for implementation in the wine [32] and yogurt production process [33], as well as for quality characterization purposes [34, 35]. For both e-noses and e-tongues, the deconvolution of the complex dataset is a crucial aspect involving the development and validation of modeling protocols based on supervised and unsupervised multivariate approaches.

Table 1 reports a shortlisting of studies in which the above-mentioned techniques have been implemented in-line or on-line with the process.

**Table 1:** Examples of studies dealing with in-line and on-line process monitoring in the food industry

Process Analyzer	Process interface type	Process type/monitored parameters	Data analysis	Reference method	Ref
NIR spectroscopy	In-line, immersion probe	Production of cheese/control of milk renneting	PCA- Hotelling’s T^2^	Rheological measurements	[18]
Vis/NIR full-transmittance spectroscopy	In-line, non-contact	Apples on a conveyor unit/detection of watercore defect	LS-SVM; one-way ANOVA of two band-ratio	Apple inspection according to US Department of Agriculture	[23]
Laser-induced fluorescence spectroscopy	In-line, non-contact	Pistachio kernels on a conveyor unit/determination of AFB1 contamination	PLS	Artificially contaminated kernels	[26]
Raman spectroscopy	In-line, non-contact	Yogurt fermentation/determination of protein, fat, sucrose, and total solids content	PLS	People’s Republic of China national standard methods	[18]
Raman spectroscopy	In-line, non-contact	Ground salmon and mechanically recovered ground chicken on conveyor unit/concentration of the fatty acids EPA + DHA (in salmon) and ash concentration (in chicken)	PLS	Gravimetric analysis; AOCS Official Method Ce 1b-89	[25]
NIR spectroscopy/electronic nose	In-line (NIR), on-line (EN)	Yogurt and Filmjölk fermentations/monitoring of the process and determination of pH and titratable acidity values	PCA, PLS	Portable pH meter, titration	[20]
Electronic nose, MOS sensors	On-line	Dough fermentation and wheat bread baking/aroma monitoring for identification of the type and stage of the process	PCA	GC–MS	[31]
Electronic tongue, voltammetric sensors	In-line	Dairy production/monitoring of changes of incoming sources of raw milk and of cleaning procedure	PCA	-	[35]

*LS-SVM*, least squares-support vector machine; *ANOVA*, analysis of variance; *PCA*, principal component analysis; *PLS*, partial least square; *EN*, electronic nose; *MOS*, metal oxide semiconductor; *EPA*, eicosapentaenoic acid; *DHA*, docosahexaenoic acid

### Requirements for the successful application of PAT

In the context of PAT applications, it is necessary to bridge the gap between the many promising scientific studies and the effective implementation of these strategies in the food industry. Currently, published applications on promising approaches for implementing PAT in the food sector are mostly limited to the laboratory scale, and predictive models are often tested with off-line measurements, as they are not directly transferable to an industrial scale.

In general, the requirements of process analysis far exceed those of laboratory-based analytical methods, even for the simplest applications. Indeed, a fit-for-purpose process analytical strategy must provide reliable quality data even under routine plant operating conditions and with negligible operator and expert intervention. PAT analyzers should maintain their operation autonomously, for example, by self-maintenance and self-calibration. In addition to robust process instrumentation with adequate analytical performance, PAT solutions require suitable instrument validation and compliance, as well as a comprehensive program of on-site process instrument (metrology, instrument maintenance, training, sufficient on-site instrument specialist), identified performance metrics, and continuous improvement plans [36].

On an industrial scale, probes have to be scaled up, and sampling systems and analyzers have to maintain robust performance under harsh and more complex operating conditions as high temperatures or probe fouling. This challenges system robustness and requires solutions to ensure constant quality and regularly recalibrate the analyzer systems without negative impacts due to process interruption. Since the PAT measures the key quality indicators of raw and processed materials as well as the key process indicators in real time and data play a central role in establishing effective performance of the PAT tools, the quality of the measurement is closely linked to the performance of a monitoring and control system. For instance, issues such as equipment window fouling or probe fouling of in situ NIR probes can occur, leading to biased spectra and misinterpretations. Therefore, the conditions of in-process spectroscopy have to be distinguished from the world of the well-conditioned laboratory, necessitating the development of sophisticated sensors and new robust chemometric calibration methods to address these problems.

Efforts in the development of PAT tools are also related to the fact that a prediction model developed for an analytical instrument may not be directly applied to other similar instruments due to variations in instrument components and the measurement environment [5]. As addressed by Müller-Maatsch et al. [37], more efforts are needed to support the portability of spectral databases between updated and previous versions of the device hardware, as well as between new and previous optical devices used for rapid on-site analysis. Without this and without the often necessary recalibration of the measuring system, the application of rapid portable devices and real-time PAT monitoring and data analysis is problematic.

## Rapid screening methods for PON testing

Screening methods that can provide quick responses on qualitative or semi-quantitative results are very important for PON food testing, being affordable, sensitive, user-friendly, rapid and robust, equipment-free, and deliverable to end users. Screening methods do not provide quantitative responses, but rather binary results in the form of yes/no answers, or positive/negative results, indicating only the detection or not of an analyte above a certain concentration level.

According to the current EU regulation, the result of the screening analysis should be reported as “negative” or “suspect.” Suspect samples in screening tests used for official controls have to undergo a follow-up analysis using a confirmatory method that can unequivocally identify and quantify the substances in order to declare whether the sample is truly non-compliant or compliant [38, 39]. These requirements are not applied for PON devices used for applications different from official controls, thus requiring a deeper insight into the concept of fitness for purpose especially when complex matrices like food are considered.

### Analytical devices for PON testing

**Fig. 3:**
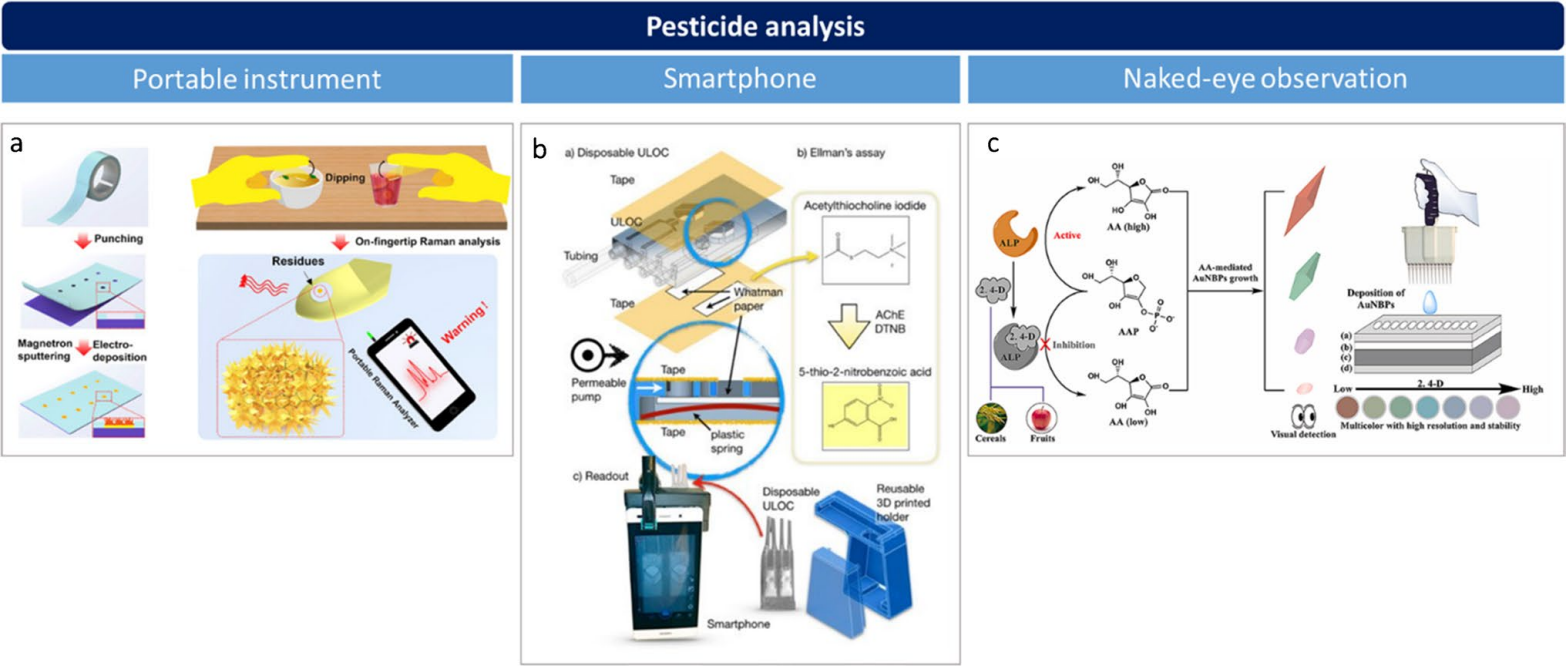
PON devices for the analysis of pesticides in food involving portable instrument, smarthphone, or naked-eye readout; **a** surface-enhanced Raman scattering (SERS) assay, based on microdroplet-captured tapes, that can be carried out on the subjects’ fingertip (lab-on-a-glove). Reprinted with permission from [47]; **b** lab-on-a-chip biosensor using 3D-printed paper and smartphone-based colorimetric detection. Reprinted with permission from [46]; **c** paper-based multicolor sensor exploiting gold nanobipyramids for naked-eye observation. Reprinted with permission from [48]

A wide variety of PON devices have been recently developed or even commercialized, most of which are based on sensors for screening tests because of their convenience in terms of rapidity, simplicity, in situ detection, and cost-effective analysis. They vary according to the type of sensors and readout: they can be based on biological and biomimetic receptors (e.g., antibodies, aptamers, oligonucleotides, enzymes) as well as on chemical receptors responsible for the interaction with the target analytes, being optical or electrochemical [40] transduction techniques generally used. The readout could involve the use of portable instruments, a smartphone, or visual (naked-eye) observation, as shown in Fig. 3 for pesticide analysis. Applications include the detection of allergens [41–43], mycotoxins [44, 45], pesticides [46–48], antibiotics [49, 50], and pathogens [51–53]. Examples of novel PON devices developed for the detection of these contaminants and other target substances in various food matrices are listed in Table 2 [41–71]. In the case of studies reporting a comparison with an independent reference method, agreement of qualitative [41, 42] or quantitative results [48, 50, 66, 67] between the two methods was observed.

**Table 2:** Examples of recent studies on the detection of allergens, mycotoxins, pesticides, antibiotics, and other target substances in various food matrices applying novel PON tests

Detection method	Analyte	Real sample/spiked matrix	Qualitative/ quantitative purpose	Comparison with an independent reference method	Investigated performance parameters	Ref
AuNPs-coated LFIA strips with colorimetric gluten detector using linear-array camera	Gluten	Flours, processed foods	Qualitative	Yes, ELISA kits	STC, LOD, false negative rate, false positive rate, cross-reactivity, recovery rates, precision	[41]
Electrochemical sensor based on MIP-modified screen-printed electrodes	Soy allergen proteins	Different processed foods	Qualitative	Yes, commercial soy LFIA Kit	LOD, cross-reactivity	[42]
Naked-eye multiplex colorimetric LFIA based on AuNP and AgNP for food allergens detection	Casein, albumin, hazelnut protein	Biscuits	Qualitative	No	LOD	[43]
Smartphone sensor based on MIP membranes and fluorescence detection	Zearalenone	Rye, wheat, maize flour	Qualitative and quantitative	No	LOD, cross-reactivity	[44]
Paper-based strips functionalized with gold nanobipyramids with portable colorimeter readout	2,4-dichlorophenoxyacetic acid	Rice, apple	Quantitative	Yes, HPLC–MS/MS	LOD, selectivity, recovery rates	[48]
Smartphone-based QD ratiometric fluorescence-sensor for visual detection on paper substrate	Gatifloxacin	Milk, drinking and fish farming waters	Qualitative and quantitative	Yes, HPLC (no information about detector)	LOD, recovery rates, selectivity, repeatability	[50]
Multicolor electrochemiluminescence biosensor with bipolar electrode, immunomagnetic separation, and colorimetric naked-eye detection	Salmonella typhimurium	Milk, beef, and other processed foods	Qualitative and semi-quantitative	No	LOD, cross-reactivity	[51]
Microfluidic AuNP-based immunosensor with colorimetric detection carried out by smartphone imaging APP	Escherichia coli O157:H7	Spiked chicken	Quantitative	No	LOD, cross-reactivity	[52]
Multiplex lateral flow immunoassay based on red and blu AuNPs based on visual readout and smartphone detection	Aflatoxin B1, type-B fumonisins	Wheat flours, cereal-based processed foods	Qualitative and semi-quantitative	No	LOD, cut-off level, false negative rate, false positive rate, precision, accuracy	[54]
SERS using a filter paper and AuNP-based substrate and portable Raman spectrometer	Methyl parathion	Apples	Quantitative	No	LOD, recovery rates, precision	[60]
Smartphone biosensor based on LFIA with chemiluminescence detection	Ochratoxin A	Red and white wine, instant coffee	Quantitative	Yes, HPLC-FLD	LOD, detection capability (CCβ), cut-off level, cross-reactivity, recovery rates/trueness	[66]
Microfluidic colorimetric immunoassay based on AuNPs and magnetic nanoparticles with UV spectroscopy and smartphone detection	Alternariol monomethyl ether	Cherry, apple, orange	Quantitative	Yes, HPLC–MS	LOD, cross-reactivity, recovery rates/trueness, precision	[68] [67]
Smartphone-integrated printed paper sensor and colorimetry based on Cu@Ag NPs	Dimethoate	Tomatoes, radish	Quantitative	No	LOD, selectivity, recovery rates/trueness, precision,	[68]
Smartphone aptasensor based on Li^3+^- assisted AuNP aggregation	Chloramphenicol	Milk, chicken	Quantitative	No	LOD, recovery rates, cross-reactivity	[69]
Colorimetric fluorescent sensor based on paper strips and smartphone readout	Cadmium	Rice	Quantitative	No	LOD, selectivity, recovery rates/	[70]
Signal-enhanced LFIA based on tyramine-induced AuNPs aggregation with naked-eye detection and quantification with portable reader	Danofloxacin	Chicken and pork meats	Qualitative and quantitative	No	LOD, cut-off level, cross-reactivity, recovery rates, precision	[71]

*LFIA*, lateral flow immunoassays; *AuNPs*, gold nanoparticles; *AgNPs*, silver nanoparticles; *NC*, nanocellulose; *QDs*, quantum dots; *SERS*, enhanced Raman scattering spectroscopy; *MIP*, molecularly imprinted polymer; *STC*, screen target concentration; *HPLC*, high-performance liquid chromatography; *FLD*: fluorescence detection; *MS*, mass spectrometry

Application of test kits with a visual readout of results based on color changes has expanded to multiple areas where rapid tests are required, the most popular being the LFIA (lateral flow immunoassay), based on immunochromatographic test strips [43, 54]. LFIA is a low-cost paper-based platform for the detection and determination of analytes in complex mixtures, where the sample is placed on a test device and the outcomes are displayed within a few minutes. However, while allowing for fast and low-cost analysis without requiring expertise from users, visual detection methods suffer from low sensitivity and are limited to qualitative and semi-quantitative analysis.

Novel applications provide an even more user-friendly design through the incorporation of LFIA test strips together with the necessary reagents in a single disposable cartridge exploiting detection by portable miniaturized colorimeters [43, 55].

In addition to portable colorimeters, portable optical detectors for rapid PON analysis include miniaturized NIR spectrometers, which are used for the development of simple, non-invasive, and cost-effective methods for qualitative or semi-quantitative applications such as the assessment of egg freshness [56] and meat adulteration [57]. Portable Raman spectrometers are also commercially available, albeit at a significantly higher cost, aimed primarily at food operators for on-site or on-field analysis. As an alternative spectroscopy technique, surface-enhanced Raman scattering (SERS) has demonstrated a great potential for rapid detection of a number of food contaminants including heavy metals, agricultural and veterinary drug residues, foodborne pathogenic microorganisms, offering the advantages of high sensitivity and selectivity, non-destructive nature, and significant improvement in the identification of the target analytes [58–60].

Portable electrochemical detectors offer great potential for quantitative PON food analysis although fewer applications can be found than optical and colorimetric detectors [42, 61]. A novel electrochemical analyzer described for the first time by Lin et al. [62] has been already commercialized as a portable gluten detector for consumers [63]. The device enables the quantitative analysis of various allergens in different foods using disposable cartridges and integrated grinding system.

In the context of PON analytical devices, the emerging smartphone-based sensing methods could revolutionize the food testing concept by making it widely accessible to small farmers, food suppliers, and even consumers. Smartphone-based colorimeters with dedicated mobile applications for the semi-quantitative or quantitative interpretation of results are a very popular low-cost alternative to commercial colorimetric readers, for example, for the readout of LFIA strips [49, 54, 64]. The popularity and portability of smartphones along with the high imaging resolution of the integrated cameras, fast processors, and customized apps for interpreting results make them ideal portable analyzers [65]. In addition, the real-time data collection and processing can be achieved by applying the IoT and machine learning algorithms. For more robust measurements, 3D-printed sample holders that can be attached to the camera or special boxes that can limit the influence of ambient light have also been developed [66].

Very recently, Jafari et al. [72] evaluated the extent to which available and novel portable devices for PON analysis fulfil the World Health Organization's ASSURED criteria: affordable, sensitive, specific, user-friendly, rapid and robust, equipment-free, and deliverable to end users. By equally weighting all the criteria, they have ranked paper-based optical and smartphone-based optical devices as the most promising for PON analysis although their sensitivity was the lowest compared to smartphone-based electrochemical or microfluidic chip–based devices. As pointed out by the authors, an important challenge for novel PON applications is related to the need to homogenize the samples, in particular for solid foodstuffs, in order to obtain representative results. In this context, more efforts are needed to demonstrate the fitness for purpose of PON devices for more “challenging” heterogeneous and solid food matrices, while maintaining rapid and user-friendly features.

Based on these considerations, it should be noted that the association of smartphones with portable analytical devices that can be used in decentralized contexts presents critical aspects depending on the information read and processed. In particular, the use of the smartphone camera to read the test and control lines of the LFIA tests intended for naked-eye detection can be considered reliable when the test is aimed at obtaining a qualitative yes/no answer, but it certainly cannot provide quantitative results based on the color intensity of the lines. As for LFIA tests based on fluorescent quantum dots (QDs), the use of smartphones to perform quantitative analyses presents critical issues that affect data reliability, when compared to the performance of portable fluorimeters. In fact, the reading of light output through smartphone cameras suffers from standardization problems caused by inter-phone variance, RGB color channel choices, and lighting options. In this respect, most of these systems use light-shielding boxes to compensate for measurement errors due to the variation of the background illumination, but just as many are based on direct reading without a box. These differences result in large fluctuations in analytical parameters such as detection (LOD) and quantitation (LOQ) limits and sensitivity depending on the smartphone used and the reading setup [73].

In general, the main standardization needs referred to real-time tools for food analysis are related to the measurement reliability and the validation of the devices with the evaluation of their performances. In some cases, challenges are already associated with the definition of the measurand, and furthermore the quantification of the measurement uncertainty associated with the application of sensor systems is particularly critical.

As for the validation of screening methods, only a few studies include all the method performance characteristics, i.e., LOD, detection capability, calibration range, repeatability, specificity, and stability. Instead, published articles focus primarily on reporting the LOD of the assay, thus ignoring the other key performance parameters [73]. As highlighted by Tsagkaris [40], more efforts are needed for validation and benchmarking of screening methods, especially of smartphone-based methods, to avoid false negative results and ensure that the methods are fit for purpose. It is worth underlining that the highlighted limitations are counterbalanced by the performances of the PON devices in terms of high throughput, rapid response, and simplification of sampling and sample treatment. This resulted in a progressive move towards smart sampling for food safety, quality, and fraud control, although these handheld devices come with drawbacks, including lower performance compared to high-end laboratory equipment [74]. The same authors highlighted how the development of these decision support tools (DSTs) will ultimately move the first line of analytical defense from laboratories to the food manufacturing sites, allowing for risk-based sampling that will help identify quality issues and adulteration at an earlier stage than is possible now. A crucial aspect that allows the application of such devices is their validation for the type of analysis or screening aimed at demonstrating the fitness for purpose in the environment in which they will be used [75].

Another distinctive aspect that affects the usability and reliability of portable devices concerns the data transmission protocol on the smartphone. Many electrochemical sensing devices use a Bluetooth® connection with the smartphone, but this requires the physical proximity of the device, within a range of about 10 m, and considerable consumption of the batteries. On the other hand, the use of Wi-Fi protocols overcomes these limitations, also offering the possibility of transferring analytical data for their on-cloud storage, thus making them shareable with multiple final display devices, which can be a smartphone, a tablet, or a personal computer [76]. The advantages related to the integration of IoT systems with Wi-Fi protocols consist both in the possibility of transferring raw data that can be processed on-cloud with enormous energy savings and greater autonomy, but also and above all in the consequent traceability of data, which are stored on-cloud [76]. Even in the absence of a Wi-Fi connection at the time of acquisition of the analytical data, these are temporarily stored in the device buffer memory and subsequently transferred when the connection is available.

### Guidelines for analytical performance assessment of screening methods

In general, prior to their routine application, the fitness for purpose of analytical methods has to be demonstrated; furthermore, when using validated methods, it is recommended to ensure that acceptable performance is achieved [77]. As for screening methods, they should meet basic performance criteria prior to their being used for decision-making purposes to ensure test reliability [39]. Qualitative, semi-quantitative, or quantitative methods can be used as screening methods, with different performance characteristics to be determined depending on the type of application [39].

Different approaches to the validation of screening methods have been proposed: while the EU Regulation 2021/808 [39] provides a general guideline for the validation of screening and confirmatory methods that can be applied to residues of pharmacologically active substances used in food-producing animals, the AOAC Guideline 2014 [78] is a general guideline for the validation of qualitative binary screening methods making use of the model proposed by Wehling et al. [79].

According to the EU Regulation 2021/808, the relevant performance characteristics shall be verified during validation in line with the scope of the method. In the case of screening methods, this shall at least include (i) the detection capability (CCβ), e.g., the smallest concentration that can be detected by the method with an error probability of β (false compliant decision), (ii) selectivity/specificity, e.g., method’s capability to distinguish between the analyte and other interfering substances, (iii) ruggedness/robustness, and (iv) precision, if screening methods are used for quantitative analysis. Furthermore, certified reference materials (CRMs) should be the first option to determine the trueness of a quantitative screening method; however, in the absence of a suitable CRM, it can be acceptable to determine the recovery rates of a known reference analyte added to the sample.

According to the AOAC guideline, qualitative methods used to make a detection decision by comparing the value of a response with a cut-off value should be validated using quantitative statistics on responses wherever possible [78]: for this purpose, a probability of detection (POD) model is proposed based on single-laboratory studies, i.e., selectivity and matrix studies, and on a collaborative study to also characterize reproducibility of the candidate method among testing laboratories. Despite the use of RMs in the examination of nominal properties has been specified even in their official definition (International Vocabulary of Metrology) since many years, their application in qualitative analyses remains challenging and there is a lack of common guidance on the production of RMs for nominal properties, as well as the approaches and understanding of terms properly defined for quantitative properties (e.g., homogeneity) are differently interpreted and applied by the various organizations and bodies.

Screening methods for selected food contaminants have also been regulated, as in the case of the EU Regulation No 519/2014 [38], which establishes the criteria for the validation and verification of qualitative and semi-quantitative screening methods for the detection of mycotoxins, and the 2010 EURLs Guideline, recently revised according to Regulation 2021/808, on validation of screening methods for residues of veterinary residues [80].

### Additional concerns about food testing carried out by non-qualified personnel

So far, the responsibility for the quality and safety assurance of food products has been entrusted to food producers, farmers, food manufacturers, and suppliers, while food control authorities are tasked with controlling compliance with legislation standards. Consumers are only expected to trust that the food they purchase is safe and that it corresponds to the described product characteristics. In recent years, though, consumers and food distributors have tended to seek more information, and this has been driving research towards widely accessible devices that can offer fast answers through simple analytical procedures.

It should be noted that the number and complexity of food matrices create a demand for properly validated testing devices with comprehensive user instructions (definitions of technical terms can be found in ISO 5725–1:1994 and the International Vocabulary of Metrology). This is crucial, for example, with food allergen determinations that can have potential lethal consequences. In this context, voluntary guidelines for consumer food gluten and allergen testing devices have recently been established, to ensure that non-qualified personnel are provided with sufficient information to make an informed decision based on an analytical result from a PON device [81]. The guidelines are based on currently known technologies, analytical expertise, standardized AOAC INTERNATIONAL allergen community guidance, and best practices on the analysis of food allergens and gluten. To establish the fitness for purpose of consumer analytical devices, they recommend a sequence that includes both single-laboratory validation and participation in external quality assessment schemes. In the case of PON analytical devices, proficiency testing schemes should include consumers or untrained personnel as testers [81].

In the case of PON testing, the assessment of the analytical performance of the device is not enough for the fitness-for-purpose evaluation, since the results are also affected by the variability of the environmental conditions together with non-technically qualified users. Sampling issue is another aspect to consider, especially when it is performed by non-expert personnel, since it is a complex process depending on the matrix to be analyzed with issues related to the size, homogeneity, and representativeness of samples. Provisions for sampling are often provided by the current legislation especially when official controls have to be performed, but the sampling of small test portions to be used for miniaturized high throughput methods is still a debated subject. In this context, in the work by Zhang et al. [41], dealing with the development of a handheld consumer gluten detector, important issues have been raised regarding the weight and inhomogeneity of the food sample; unlike laboratory procedures where sample weight can be measured and large sample can be properly ground and homogenized, the reliability of consumer testing must rely on user education even though sampling still remains a great issue. Despite these critical issues, Kalinowska et al. [65] highlighted the drastic improvement that the implementation of smartphones could bring in food control, especially in developing countries where access to conventional instrumental methods can be limited and expensive, emphasizing in particular, the beneficial use of smartphone-based PON devices for in-field monitoring of products by farmers and small producers in remote areas, as part of a prevention strategy for foodborne illness caused by food contaminants.

## The need for metrological support to PAT strategies and PON devices

PAT strategies and PON devices are under development, which partly explains why many researchers publish results without necessarily providing metrological support and demonstrating adequate information on performance characteristics. This makes the reliability of such methods rather difficult to prove and comparability with confirmatory methods challenging [40].

Many international organizations and committees are striving to improve the consistency of results among laboratories in general such as the International Organization for Standardization (ISO) [82] or the European Committee for Standardization (CEN) [83] and in specific fields like food safety and security the Codex Alimentarius [84], the Codex Committee on Contaminants in Food [84], and the European Union Reference Laboratory for Genetically Modified Food and Feed (EURL-GMFF) [85]. One of the international groups dedicated to improving the comparability of measurements is the Working Group on Food Safety, Trade, and Authenticity of the Consultative Committee for Amount of Substance; Metrology in Chemistry and Biology (CCQM). As established by the CCQM, the strategy to be followed in the period 2021–2030 is aimed at improving the international comparability of chemical and biological measurements so as to enable the Member States and Associates to perform measurements with a high confidence level [86]. Food safety, trade, and authenticity is one of the nine sectors that are expected to influence the CCQM strategy, which recommends the use of suitable metrological tools to ensure food safety and authenticity.

Metrological traceability of measurements to the International System of Units (SI) is essential for ensuring their comparability both at a national and international level. As stated in a previously published review article on this topic [1], given the huge demand for comparability of analytical results because of the globalization of food trade and national and international regulations on food safety, the objectives of ensuring an adequate level of food quality and safety can be pursued by implementing quality assurance measures along the entire food chain, but also through the use of validated analytical methods and the accreditation of testing and calibration laboratories according to the international standard ISO/IEC 17,025 [87]. On the other hand, there is also the need for the development of real-time monitoring methods for food process control, since the food industry is responsible for setting up food safety management systems that deliver foodstuffs in compliance with the legislation. In such cases, reliable data derived from validated analytical methods are needed to enable industry stakeholders and regulators to make sound scientific decisions.

The metrological traceability of measurement results requires an unbroken chain of calibrations of the measuring device to references all having stated measurement uncertainties [88]. RMs and CRMs together with method validation, proficiency testing, and official controls represent the main tools to address metrology for food safety [1, 39, 89–91].

In a recent review paper devoted to discussing recent advances in miniaturized analytical tools for mycotoxin detection and the challenges related to point-of-need analysis, Soares et al. [92] pointed out that progress is needed to make analytical instruments portable with poor sample preparation, but also highlighted the need to follow standardized procedures for evaluating the performance of such methods, including the use of reference materials. The lack of RMs and in particular of matrix-RMs for food analysis, in particular for some matrix-analyte combinations, represents a bottleneck for the validation of methods that requires the use of alternative approaches such as real samples spiked with the analytes of interest [93].

Furthermore, there is still a lack of use of RMs for qualitative analyses, taking into account that the need for guidance documents for the production of RMs certified for nominal properties has been recognized by many experts. Even though there is a lack of an internationally harmonized guidance document, ISO/TR 79:2015 aims to contribute to the on-going discussion on nominal properties and the production of such RMs. Therefore, less attention is paid to metrological support of results when assessing qualitative methods, since the definitions of accuracy, uncertainty, and precision cannot be applied in the same statistical context as quantitative analysis. This makes the metrological approaches used to demonstrate the analytical reliability of rapid screening tests rather inconsistent [91, 94], while on the other hand there is an urgent need to assure the quality of the results provided by rapid, miniaturized, simple, and direct analytical processes. In addition, effective process monitoring and real-time corrections will strictly depend on the accuracy and traceability of the measurement that feeds the data into the automatic system control. In fact, metrological traceability represents a prerequisite not only for the reliability of measurement data for process control in the plant, but also for ensuring a product quality that is mutually recognized in the global food market.

**Fig. 4:**
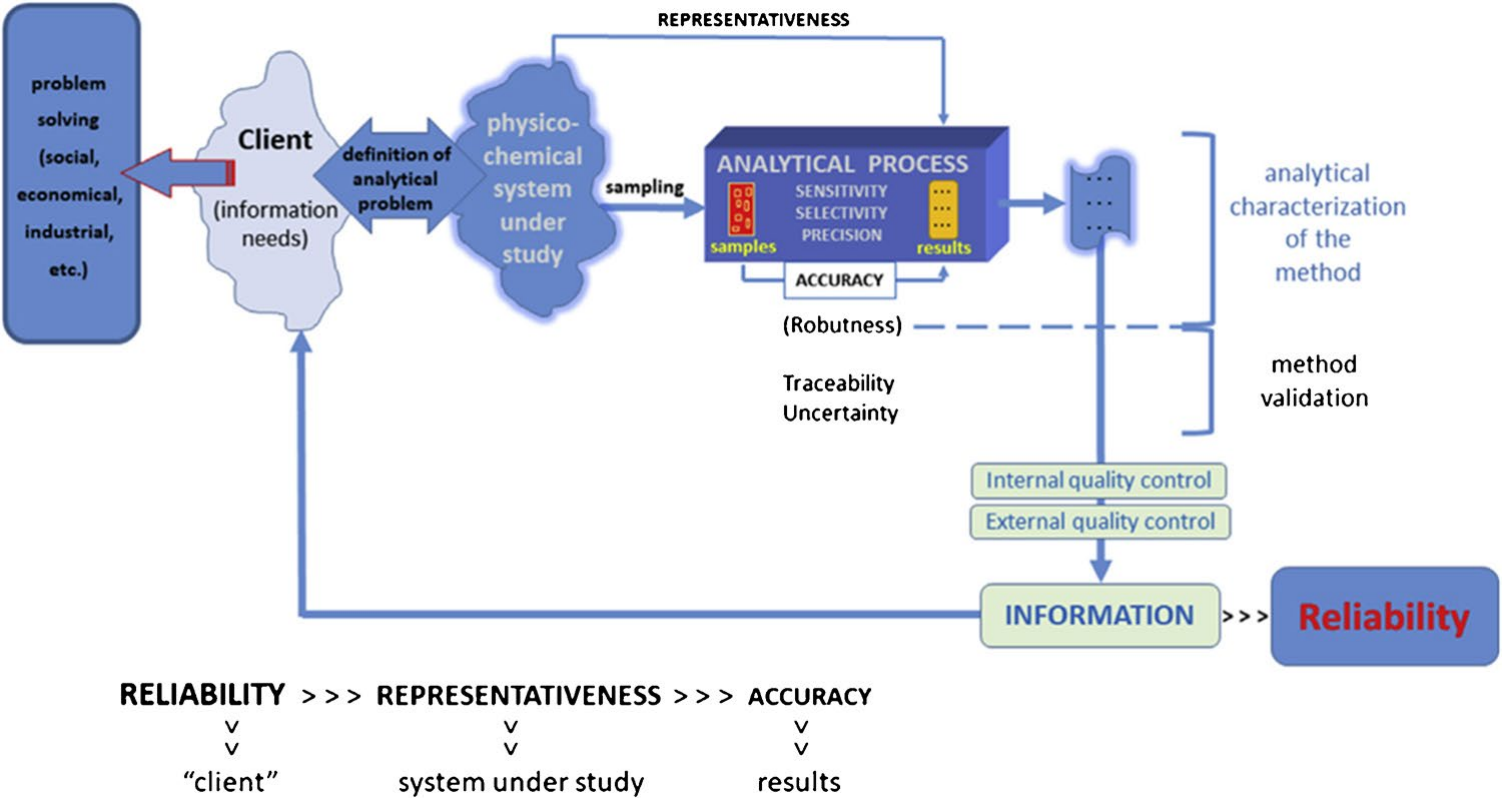
Different hierarchical levels between analytical information and the client information needs. Reprinted with permission from [91]

In this context, Soriano et al. [91] proposed the use of analytical reliability as a summative property for methods providing qualitative and quantitative conventional results, total indices, and method-defined parameter, i.e., a measurand that can be obtained only by using a well-established (bio)chemical measurement process. They also recommend introducing reliability studies in the first part of the validation processes of the method because of the added value of this property in the characterization of an analytical method, including both quality of the analytical information and user needs (Fig. 4).

Most of the PON devices have not been validated or their operation has not been evaluated with the sensitivity required for “real-life” conditions, i.e., they have been tested with concentration levels above the current maximum regulatory limits of the analytes [74]. These aspects hamper performance comparability of PON devices as well, which often are still at the proof-of-concept stage. This situation applies also to some published PAT studies, where the performances of an in-process monitoring strategy, associated with a proper calibration or classification model, are assessed in an off-line mode, i.e., by using benchtop instrument on grab samples, properly pre-treated, from different process stages [27, 95]. Of course, it is necessary to go beyond the proof-of-concept and feasibility studies in order to prove the actual reliability of the PAT strategy in real plant conditions.

The effective implementation of PAT and in-process measurements relies on the selection of a process analyzer and its positioning for plant-wide process monitoring. As discussed by van den Berg et al. [96], a rational decision on this matter is made particularly difficult due to the incomparability of different instrument characteristics; in fact, as claimed by the authors, a fast but imprecise instrument is incomparable to a slow but precise instrument. For this reason, van den Berg and coworkers [2, 96] proposed an objective assessment of the performance of process analyzers through the calculation of a quantitative factor for which uncertainty of measurements plays a key role in combination with measurement frequency, grab size, carry-over effect, and delay time between taking the sample and obtaining the result.

Finally, as already observed, since PON devices are not designed to be used by qualified laboratories, the responsibility for validating and testing their performance for their intended use rests with the manufacturers of the device. Both the scientific community and technology developers should be encouraged to make additional efforts to follow standard procedures for the validation and verification of the methods’ applicability in real-life conditions, as in the case of the AOAC Guidance document for consumer analytical device on gluten and food allergens [81]. In the aforementioned AOAC document, on the other hand, PON devices are enabled as DSTs also for end users, in order to make these devices usable for the evaluation of the safety of foods labelled as gluten-free. For this reason, it is important to raise the awareness of PON device manufacturers to invest in rigorous validation to maximize their analytical robustness, especially with regard to sampling and sample handling procedures.

**Fig. 5:**
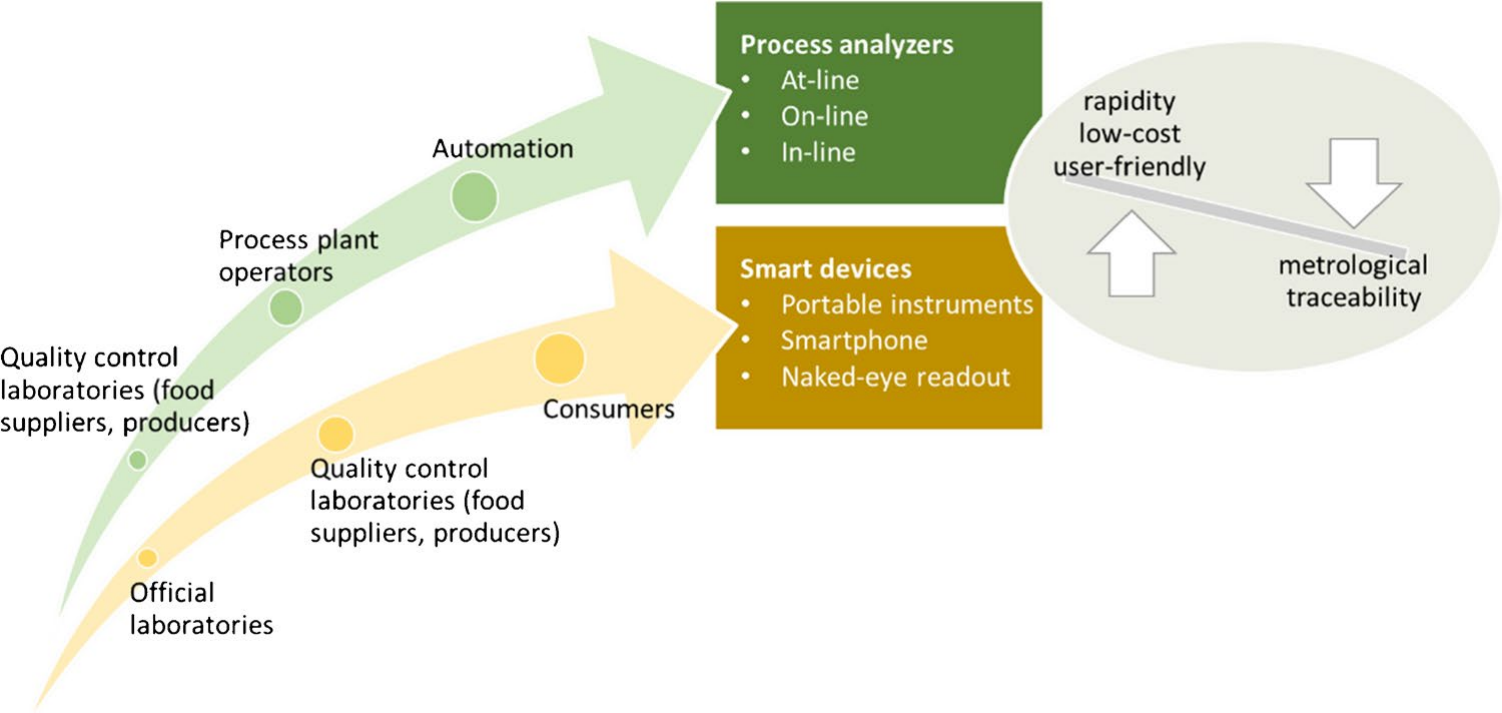
Keeping the balance between the desired performance characteristics of PAT-based processes and PON tests and metrological traceability

In this context, the reliability of the measurement results and the metrological support for PON devices and PAT analyzers for real-time and on-site analysis, especially commercial ones, should be in balance with the well-known advantages in terms of rapidity, cost-effectiveness, ease of use, so that their use can be fit for purpose (Fig. 5). This balance is often not respected as researchers describe innovative methods without simultaneously reporting information on their performance characteristics to evaluate the quality and reliability of the analytical data for the intended use.

## Conclusions

The development of reliable methods for portable easy-to-use point-of-need screening devices has considerable potential for testing food quality and safety even by consumers who, no matter where they are, can receive quick and accurate information about the food they are eating. At the same time, the development of reliable methods for real-time monitoring and process control has considerable potential for testing food quality and safety in food production, where getting accurate results quickly can save money and reduce the risk of recalls to a minimum. In this context, metrological reliability plays a key role in ensuring the comparability of analytical results. This requires the attention of the research community and devices manufacturers to ensure the reliability of measurement results from PAT strategy and PON tests through the critical assessment of performance characteristics. The ethical issues related to the involvement of consumers as the final link of the measurement chain still represent an open issue: educating the population in the correct use of these smart devices and how to behave in relation to non-compliant results will be a major challenge of the next future.
